# Anemia and associated factors among school-age children in Filtu Town, Somali region, Southeast Ethiopia

**DOI:** 10.1186/2052-1839-14-13

**Published:** 2014-08-18

**Authors:** Bekele Gutema, Wondimagegn Adissu, Yaregal Asress, Lealem Gedefaw

**Affiliations:** Department of Clinical Laboratory, Filtu District Hospital, Somali region, Somali Ethiopia; Department of Medical Laboratory Science and Pathology, College of Public Health and Medical Sciences, Jimma University, Jimma, Ethiopia

**Keywords:** Anemia, Associated factors, School-age children, Somali region

## Abstract

**Background:**

Anemia is one of the major public health problems affecting more than half of school-age children in developing countries. Anemia among children has been conclusively seen to delay psychomotor development, poor cognitive performance, impaired immunity and decrease working capacity. The purpose of this study is to determine the prevalence and associated factors of anemia among school-age children in Filtu Town, Somali region, Southeast Ethiopia.

**Methods:**

A community based cross-sectional study was conducted from July to August, 2013 in Filtu Town. A total of 355 school-age children between 5–15 years old were included in the study. Socio-demographic data were obtained from each participant using structured questionnaire. Hemoglobin concentration was determined by HemoCue 201^+^ photometer (HemoCue, Angelholm, Sweden) analyzer. Hemoglobin values below 11.5 g/dl and 12 g/dl were considered as anemic for age ranges of 5–11 and 12–15 years, respectively. Anthropometric data were taken from each study participant. Peripheral blood film and stool examination were done for hemoparasite and intestinal parasite screening, respectively. Data were analyzed using SPSS version 16.0.

**Results:**

Over all, prevalence of anemia was found to be 23.66%. The vast majority (73.81%) of the anemic children had mild anemia. Moderate and severe anemia accounted for 25% and 1.19% of the anemic children, respectively. Being from a family with low income (AOR = 9.44, 95% CI: 2.88, 30.99), stunted (AOR = 5.50, 95% CI: 2.83, 10.72), underweight (AOR = 2.07, 95% CI: 1.06, 4.05) and having intestinal parasite infection (AOR = 2.99, 95% CI: 1.05, 8.46) were identified as associated factors for anemia.

**Conclusion:**

Anemia is a moderate public health problem in school-age children for the study area. Interventions targeting nutritional deficiencies and parasitic infections are recommended.

## Background

Anemia is a condition which results from a reduction in hemoglobin concentration or reduction in red blood cell number or both resulting in lower ability of oxygen delivery to support the body’s activities. Anemia is a public health problem in developing countries among school-age children. The highest prevalence of anemia exists in the developing world where its causes are multi-factorial. In the developing world, the prevalence among school-age children ranges from 12–60% [1–3]. Although the national level prevalence of anemia in Ethiopia is considered to be mild (with prevalence of 5–19.9%), some regions exhibited high prevalence of anemia. According to the Ethiopian demographic and health survey report for the year 2005 and 2011, unlike most regions in the country, Somali region did not show a significant decline in the prevalence of anemia in the previous five years [4, 5].

Anemia in children may resulted from poor bioavailability of iron, infections like intestinal parasites, malaria and tuberculosis (TB). Although anemia has a variety of causes, it is generally assumed that 50% of cases are caused by iron deficiency. The main risk factors for iron deficiency among young children in developing countries are malnutrition (low intake) and high requirement of iron during child growth. Due to the multifactorial conditions, complexity of risk factors of anemia and potential interactions among them, a single strategy to control anemia in developing countries may have little success. An integrated strategy for anemia control and prevention is required [1, 6–11].

Anemia is one of the most widespread public health problems, especially in developing countries with greater risk in schoolage children. Anemia in schoolage children result in lowered resistance to disease, increased susceptibility to infection, poor cognitive development, impaired physical development, poor school performance and reduced work capacity with impaired social and economic development of the country [12, 13].

In Ethiopia, studies on anemia often focus in pregnant women and under five years of age children. There are limited data on anemia in school-aged children and predisposing factors. Hence this this study is conducted to determine the magnitude of anemia and its associated factors among school-age children in Filtu Town, Somali Region of South-East Ethiopia.

## Methods

### Study setting and sampling

A community based cross sectional study was conducted from July to August 2013 in Filtu Town. The town is in Ethio-Somali region, South East Ethiopia. 355 school-age children were determined using single population proportion statistical formula by the following assumptions: 95% level of confidence, 5% margin of error and P (proportion) of 0.3 taken from a study done in neighboring Somalia [14]. Systematic sampling technique was applied by using house number as sampling frame obtained from Filtu Town health extension workers office. The town has three kebeles, the smallest administration unit in the country, named as: 01, 02 and 03 with the total household of 295, 209 and 197, respectively. First, the total sample size was proportionally allocated to the three kebeles. Then, proportionally allocated numbers of households were selected by systematic random sampling method from each Kebele. Finally, from one systematically selected house hold, one school-age children was recruited. Simple random sampling technique was used when more than one eligible children found in a single household whereas the next household was considered when there was no eligible child in the systematically selected household. School-aged children between 5–15 years old were included in the study whereas those children on treatment of anemia, and children with chronic disease were excluded from the study.

### Data collection and processing

Socio-demographic, socioeconomic and other related data were collected by questionnaire based interview. Hemoglobin (Hgb) concentration was determined by Hemo Cue HB 201^+^ analyzer (Hemo Cue, Angelholm, Sweden) from capillary blood sample. Capillary blood was collected by finger pricking after rubbing the fingertip with sterile cotton (immersed in 70% alcohol), and pricking it with a sterile disposable lancet. A drop of blood was allowed to enter the optical window of the microcuvette through capillary action after discharging the first drop of blood. Then microcuvette was placed into the cuvette holder for photometric determination of hemoglobin level.

School-age children with Hgb levels lower than 11.5 g/dl and 12 g/dl was considered as anemic for age ranges from 5–11 and 12–15 years old, respectively. Mild anemia was defined as Hgb concentration of 10–11.9 g/dl for 12–15 years and Hgb concentration of 10–11.4 g/dl for 5–11 years. Moderate anemia was defined as Hgb concentration between 7–9.9 g/dl and severe anemia was defined as Hgb concentration lower than 7 g/dl [1]. Both thick and thin blood films were prepared and stained for the assessment of hemoparasites. Five (5gm) of stool samples were collected from each study participant using clean and leak-proof stool cups. Stool samples were examined using both direct wet mount method and formol-ether concentration technique.

Weights were measured with children in light clothing using a portable digital scale to the nearest 0.1 kg. Calibrated, fixed base portable wooden scale was used for height measurement to the nearest 0.1 cm. Every measurement was performed three times, and the mean values were used for analysis. All the data were transformed and expressed in Z-scores and calculated using WHO anthroPlus1.0.4 statistical software packages for ages 5–19 years. The mean Z scores were calculated and under nutrition was defined for a child, who has less than −2 z-scores (−2SD) from the National center for health statistics (NCHS) median reference population values. This was used as cut-off points for determination of malnourishment.

To assure the quality of data one day training was given for data collectors and daily supervision was made during the data collection period. Standard operating procedures (SOPs) and manufacturers’ instruction were strictly followed for all laboratory activities. All laboratory reagents were checked for their expiry date. Laboratory results were recorded on standard report formats using participants’ identification number.

### Data analysis

Data were coded, cleaned prior to analysis, entered and analyzed using SPSS V16.0 statistical software. Description statistics (mean, frequencies, cross tabulation) were done to describe the study participants and determine the prevalence of anemia. Bivariate and multivariate logistic regression analyses were done to look for statistically significant associated factors of anemia. For the logistic regression analysis, initially we have observed the association of each independent variable with the dependent variable. Those variables with P value of 0.25 or less in the bivariate logistic regression and other variables with P value > 0.25 but having scientifically proved public health importance were our candidate variables for the multivariate logistic regression model. Finally, variables having P value of 0.05 or less were taken statistically significant factors for anemia.

### Ethical approval and consent

Ethical clearance was obtained from Jimma University ethical review board and permission was taken from Filtu district health bureau to conduct the study. Written informed consent was obtained from the parents/guardians. Moreover, assent portion of consent form for children greater than 7 years old were prepared and obtained. All the study participants who were found positive for intestinal, hemoparasites and anemia were treated free of charge at Filtu district hospital, Somali region southeast Ethiopia.

## Results

### Socio-demographic characteristics of study participants

A total of 355 school-age children (52.68% male were male and 47.32% female) had participated in the study. Majority of study participants, 289 (81.41%) were within the age group of 5–11 years with mean age of 8.51 ± 2.83 years. Most of the children’s mothers (79.15%) and fathers (67.32%) were illiterate (Table 1).

**Table 1:** **Associations of anemia with Socio-demographic characteristics among school-age children in Filtu Town, Somali region, South-East Ethiopia, 2013 (n = 355)**

Variables	Categories	Anemia		COR*(95% CI)	P-value
		Yes (%)	No (%)		
**Sex**	Male	52 (27.81)	135 (72.19)	Ref (1)	
	Female	32 (19.05)	136 (81.95)	0.61 (0.30, 1.01)	0.054
**Age**	5-11	75 (25.95)	214 (74.05)	2.20 (1.05, 4.70)	0.037
	12-15	9 (13.64)	57 (86.36)	Ref (1)	
**Mother’s occupations**	House wife	59 (27.19)	158 (72.81)	Ref (1)	
	Merchant	10 (14.49)	59 (85.51)	0.7 (0.28, 1.77)	0.461
	Governmental employee	3 (10.34)	26 (89.66)	0.5 (0.10, 2.61)	0.413
	Private employee	3 (25.00)	9 (75.00)	0.9 (0.13, 5.22)	0.849
	Daily laborer	6 (31.58)	13 (68.42)	1.5 (0.44, 5.15)	0.508
	No job	3 (33.33)	6 (66.67)	0.9 (0.13, 6.87)	0.941
**Father’s occupation**	Farmer /pastoralist	28 (36.36)	49 (63.64)	Ref (1)	
	Merchant	11 (17.74)	51 (82.26)	0.38 (0.17, 0.84)	0.017
	Governmental employee	9 (13.64)	57 (86.36)	0.28 (0.12, 0.64)	0.003
	Private employee	7 (17.50)	33 (82.50)	0.37 (0.15, 0.95)	0.038
	Daily laborer	20 (24.10)	63 (75.90)	0.56 (0.28, 1.10)	0.092
	No job	9 (33.33)	18 (66.67)	0.88 (0.35, 2.21)	0.777
**Mother’s education**	Illiterate	76 (27.05)	205 (72.95)	7.4 (0.98, 56.20)	0.053
	Primary school	5 (13.89)	31 (86.11)	3.2 (0.35, 29.68)	0.301
	High school	2 (11.76)	15 (88.24)	2.7 (0.22, 32.23)	0.440
	Collage or above	1 (4.76)	20 (95.24)	Ref (1)	
**Father’s education**	Illiterate	64 (26.78)	175 (73.22)	1.9 (0.86, 4.31)	0.114
	Primary school	10 (24.39)	31 (75.61)	1.7 (0.56, 4.79)	0.320
	High school	2 (8.00)	23 (92.00)	0.5 (0.09, 2.33)	0.346
	Collage or above	8 (16.00)	42 (84.00)	Ref (1)	
**Monthly household income**	<500 ETB*	11 (50.00)	11 (50.00)	8.90 (3.43, 23.13)	<0.001
	500-1999 ETB	53 (39.26)	82 (60.74)	5.75 (3.23, 10.30)	<0.001
	≥2000 ETB	20 (10.10)	178 (89.90)	Ref (1)	
**Family size**	<5	8 (9.20)	79 (90.80)	0.26 (0.12, 0.56)	0.001
	≥5	76 (28.36)	192 (71.64)	Ref (1)	

NB: ETB* = Ethiopian Birr.

### Nutritional status and clinical characteristics of study participants

Out of all the study participants, 117 (32.96%) of them were stunted for their age, and 104 (29.30%) were underweight. Eighty one (22.82%) of the children were positive for intestinal parasites. A total of seven species of intestinal parasites were identified. *Ascaris lumbricoides* (43.21%) was predominant followed by *Giardia lamblia* (22.22%), *Hook worms* (12.35%), *Trichuris trichiuria* 8 (9.88%), *Enterobius vermicularis* 4 (4.94%), *Hymenolepis nana* 3 (3.70%) and *Entamoeba histolytica*/*dispar* 3 (3.70%). Microscopic examination of the blood films revealed 13 (3.66%) malaria cases (Table 2).

**Table 2:** **Associations of anemia with nutritional status and clinical characteristics among school-age children in Filtu Town Somali region, South- East Ethiopia, 2013 (n = 355)**

Variables	categories	Anemia		COR* (95%CI)	P-value
		Yes (%)	No (%)		
Stunting (Z score < −2SD*)	Yes	55 (47.01)	62 (52.99)	6.39 (3.76, 10.88)	< 0.0001
	No	29 (12.18)	209 (87.82)	Ref (1)	
**Underweight (Z score < −2SD)**	Yes	43 (41.35)	61 (58.65)	3.16 (2.16, 6.01)	< 0.0001
	No	41 (16.33)	210 (83.67)	Ref (1)	
**Intestinal parasite infection**	Positive	34 (41.98)	47 (58.02)	2.49 (1.14, 5.45)	0.023
	Negative	60 (21.89)	214 (78.11)	Ref (1)	
**Malaria**	Positive	7 (53.85)	6 (46.15)	4.02 (1.31, 12.30)	0.015
	Negative	77 (22.51)	265 (77.49)	Ref (1)	

COR* = Crude odds ratio, SD* = standard deviation.

### Prevalence of anemia

Overall, prevalence of anemia among school-aged children was 23.66%. Most of the anemic children (73.81%) had mild anemia. Moderate and severe anemia accounted for 25.00% and 1.19% of the cases. The prevalence was higher in males (27.81%) than the female (19.05%) children. Hemoglobin level of children ranged from 6.70 to 15.30 g/dl with mean of 12.43 ± 1.42 g/dl. Anemia prevalence was 25.95% among the age group 5–11 years old and 13.64% among 12–15 years age groups. Anemia among children whose mothers were housewives was 59 (27.19%) (Table 1).

The prevalence of anemia among stunted study participants was 55 (47.01%). Seven of the children with malaria (53.85%) and 41.98% of the children with intestinal parasitic infection were anemic (Table 2).

### Factors associated with anemia

All explanatory variables with P value of ≤ 0.25 on bivariate analysis were entered into the multivariate logistic regression models to identify independently associated factors with anemia. Accordingly, monthly household income, stunting, underweight and intestinal parasitic infection were identified independent associated factors of anemia (Table 3).

**Table 3:** **Multivariate logistic regressions of selected variables associated with anemia among school-age children in Filtu Town, Somali region, South- East Ethiopia, 2013**

Variables	Categories	COR* (95%CI*)	P-value	AOR* (95%CI)	P-value
**Family income**	<500	8.90 (3.43, 23.13)	< 0.0001	9.44 (2.88, 30.99)	< 0.0001
	500 – 1999	5.75 (3.23, 10.88)	< 0.0001	4.73 (2.31, 2.31)	< 0.0001
	>2000	Ref (1)		Ref (1)	
**Stunting (Z score < −2SD)**	Yes	6.39 (3.76, 10.88)	< 0.0001	5.50 (2.83, 10.72)	< 0.0001
	No	Ref (1)		Ref (1)	
**Underweight (Z score < −2SD)**	Yes	3.16 (2.16, 6.01)	< 0.0001	2.07 (1.06, 4.05)	0.034
	No	Ref (1)		Ref (1)	
**Intestinal parasitic infection**	Positive	2.49 (1.14, 5.45)	0.023	2.99 (1.05, 8.49)	0.040
	Negative	Ref (1)		Ref (1)	

AOR* = Adjusted Odd Ratio, CI = Confidence Interval.

Regarding monthly household income, after controlling confounding effect the odds of being anemic among children whose families monthly income < 500 Ethiopian Birr were 9.44 times higher than among children whose families monthly income > 2000 Ethiopian Birr (AOR = 9.44, 95% CI: 2.88-30.99). The odds of being anemic were 5.50 times higher among stunted children than those non stunted children (AOR = 5.50, 95% CI: 2.83, 10.72). Underweight school-aged children were 2.07 times more likely to be anemic compared to normal children (AOR = 2.07, 95% CI: 1.06, 4.05). Infection with intestinal parasite increase the likelihood of anemia by 2.99 times as compared to non- infected children (AOR = 2.99, 95% CI: 1.05, 849) (Table 3).

## Discussions

The overall prevalence of anemia among school-aged children was 23.66%, suggesting that anemia is a public health problem among the school-aged children in the area. No similar study was obtained to compare our finding with; however, 11% prevalence was reported from Northern part of Ethiopia [15]. The larger regional variation might be due to differences in geographical variation and differences in life style.

The prevalence of anemia in our study is higher than those similar studies reported from different areas like, Egyptian children 12% [16], among school-age children in Kenitra Morocco 12.2% [17] and among Sanliurfa, South-east Turkish children 5.4% [18]. This variation might be due to low socioeconomic status and lower nutritional status of school-aged children in this study area than those reported from elsewhere. For instance a study in Kenitra Morocco indicated that only 8.9% (26/293) and 12.6% (37/293) were stunted and underweight, respectively which is lower than our study where 117 (32.96%) of school-age children were stunted for their age, and 104 (29.30%) were underweight. In addition, majority of our study participants had 5 or more family members which has an effect on their quality of life.

However, prevalence of anemia in our study is much lower than similar reports conducted in Tanzania (79.6%) [19], in Kenya (35.3%) [20], in Abia State, Nigeria (82.6%) [21]. The lower prevalence of anemia in our study might be due to the fact that malaria which is one of the major causes of anemia was less prevalent (3.66%) in this study compared to the previous studies. A study in Nigeria reported that malaria parasite, which, may also contribute to the etiology, and severity of anemia through several mechanisms including destruction of red blood cells, was confirmed in majority of the children (93.2%) with *Plasmodium falciparum* as the primary cause of severe malaria (39.8%). Moreover, the prevalence of hookworm infection in our study is low and shistosomiasis is not detected, which are major causes of anemia but they were the major causes of anemia in Tanzania and Kenya [19, 20].

The prevalence of anemia changed according to socio-demographic characteristics of children and their families which showed statistically significant differences for some variables and not for others. Children from families with low monthly household income were more likely to be anemic than those children from families with high monthly house hold income (AOR = 9.44, 95% CI: 2.88, 30.99). This might be due to the fact that families with low monthly household income may not get enough iron-rich foods and diets of children living in poor families are usually monotonous. This is similar with the study conducted among students of Ningxia and Qinghai’s poor counties of rural China’s [22].

Stunted children are 5.50 times more likely to be anemic than non-stunted children (AOR = 5.50, 95% CI: 2.83, 10.72) and children who are underweight are 2.07 times more likely to be anemic than children with normal weight (AOR = 2.07, 95% CI: 1.06, 4.05). This might be due to long term effect of low iron intake and other micronutrient deficiencies. It was supported by studies conducted in Tanzania [19] reported that under weight was significantly associated with anemia (P < 0.05) by suggesting it could be diet related which is long term effect of low iron intake and Vitamin A deficiency among the children.

School-age children infected with intestinal parasites are more likely to be anemic than those none infected children (AOR = 2.99, 95% C.I: 1.05, 8.49 6.73). This is similar with the studies reported from Tanzania [19] and the study from Edo state, Nigeria [23]. This might be due to the fact that most identified intestinal parasites have their own contribution on blood loss and/or red cell destruction.

Although literature indicates there is indeed a strong association of malaria with increased prevalence of anemia through several mechanisms including destruction of red blood cells [23], we did not find an association between anemia and malaria infection probably due to low prevalence of malaria in our study area, because the present study did not consider seasonal variation of malaria.

## Conclusion

Anemia was moderate significant health problem among school-aged children with the overall prevalence of 23.66%. Malnutrition and intestinal parasitic infection are the main associated factors of anemia among school-age children. Therefore, further longitudinal studies with long term follow-up are needed to explore more on the causes of anemia among school-age children in resource limited settings for possible intervention.
